# Label-Free Quantitative Proteomics Analysis of Nasal Lavage Fluid in Chronic Rhinosinusitis with Nasal Polyposis

**DOI:** 10.3390/biology13110887

**Published:** 2024-10-30

**Authors:** Musallam Kashoob, Afshan Masood, Assim A. Alfadda, Salini Scaria Joy, Wed Alluhaim, Shahid Nawaz, Mashal Abaalkhail, Omar Alotaibi, Saad Alsaleh, Hicham Benabdelkamel

**Affiliations:** 1 Otolaryngology—Head and Neck Surgery Department, College of Medicine, King Saud University, Riyadh 11411, Saudi Arabia; mkashoob.c@ksu.edu.sa; 2 Department of Otolaryngology—Head and Neck Surgery, Sultan Qaboos Hospital, Salalah 211, Oman; 3 Proteomics Resource Unit, Obesity Research Center, College of Medicine, King Saud University, Riyadh 11451, Saudi Arabia; afsmasood@ksu.edu.sa (A.M.); aalfadda@ksu.edu.sa (A.A.A.); wedd.luhaim@gmail.com (W.A.); shnawaz@ksu.edu.sa (S.N.); 4 Department of Medicine, College of Medicine, King Saud University, Riyadh 11411, Saudi Arabia; 5 Strategic Center for Diabetes Research, College of Medicine, King Saud University, Riyadh 11461, Saudi Arabia; sjoy@ksu.edu.sa; 6 College of Medicine, King Saud University, P.O. Box 245, Riyadh 11411, Saudi Arabia; 438102891@student.ksu.edu.sa (M.A.); 438100902@student.ksu.edu.sa (O.A.)

**Keywords:** chronic rhinosinusitis, sinusitis, nasal polyposis, label-free quantification, LC-MS/MS, biomarkers, inflammatory markers, oxidative stress pathways

## Abstract

(1) Background: Chronic rhinosinusitis (CRS) is a common chronic inflammation of the nasal mucosa and the paranasal sinuses. The pathogenesis of chronic rhinosinusitis (CRS) is multifactorial and, as of yet, not well understood. (2) Methods: Nasal lavage fluid samples were collected from patients diagnosed with chronic sinusitis with nasal polyposis (CRSwNP) (n = 10) and individuals without sinusitis (control group) (n = 10) who had no nasal complaints. In the present study, we used an untargeted label-free LC-MS/MS mass spectrometric approach combined with bioinformatics and network pathway analysis to compare the changes in the proteomic profiles of the CRSwNP group and the control group. Data from LC-MS/MS underwent univariate and multivariate analyses. (3) Results: The proteomic analyses revealed distinct differences in the abundances of nasal lavage fluid proteins between the CRSwNP and control groups: a total of 234 proteins, 151 up- and 83 down-regulated in CRSwNP. Functional Gene Ontology (GO) analysis showed that dysregulated proteins were involved in airway inflammatory reaction, immune response, and oxidative stress. The biomarkers were evaluated using the Receiver Operating Characteristic (ROC) curve; an Area Under the Curve (AUC) of 0.999 (95% CI) identified potential biomarkers between the CRSwNP and control group. EMILIN-3 and RAB11-binding protein RELCH were down-regulated, and Macrophage migration inhibitory factor and deoxyribonuclease-1 were up-regulated, in CRSwNP compared to the control group. (4) Conclusions: These differentially expressed proteins identified in CRSwNP are involved in airway inflammatory reaction, immune response, and oxidative stress. In particular, the identification of increased interleukin-36 gamma (IL-36γ), which contributes to inflammatory response, and a decrease in SOD, in this group are notable findings. In the future, several of these proteins may prove useful for exploring the pathogenesis of nasal polyps and chronic sinusitis or as objective biomarkers for quantitatively monitoring disease progression or response to therapy.

## Simple Summary

This study aimed to identify proteins associated with chronic rhinosinusitis with nasal polyps (CRSwNP) using label-free quantitative proteomic analysis. Samples of nasal lavage fluid from CRSwNP patients and controls were analyzed and revealed a significant difference in protein expression: a total of 234 proteins, 151 up- and 83 down-regulated in the CRSwNP group. Dysregulated proteins were linked to airway inflammation, immune response, and oxidative stress. Several proteins, including EMILIN-3, RAB11-binding protein RELCH, Macrophage migration inhibitory factor, and deoxyribonuclease-1, were identified as potential biomarkers for CRSwNP. The findings suggest that these proteins play a crucial role in the pathogenesis of the disease and may be valuable for future research and clinical applications.

## 1. Introduction

Chronic rhinosinusitis (CRS) is a global chronic inflammatory disorder of the nasal mucosa and paranasal sinuses. Approximately 5–12% of the general population is affected by the condition, making it a significant health problem [1]. The pathogenesis of chronic rhinosinusitis (CRS) is multifactorial and, as of yet, not well understood. CRS has been simplistically classified into two phenotypes: based on the presence or absence of nasal polyps into chronic sinusitis with nasal polyposis (CRSwNP) and without nasal polyps (CRSsNP) [2]. CRSwNP represents 20–25% of cases, while CRSsNP comprises more than two-thirds of cases. Both conditions present with similar signs and symptoms, although the underlying molecular mechanisms and pathologies differ greatly. The diagnosis is based on the presence of two or more symptoms, which may include nasal blockage, obstruction, congestion, or discharge, as well as facial pain/pressure and a reduction or loss of smell. CRSwNP, in more recent times, has been considered a complex heterogeneous disease consisting of several disease variants with different underlying pathophysiologies.

CRSwNP is a heterogeneous disorder with visible polyps in the middle meatus and/or olfactory cleft with or without eosinophilic infiltration. Polyps are outgrowths of edematous inflammatory tissue that have grown into the middle meatus [3]. The underlying pathophysiologic mechanisms of developing CRSwNP need to be further elucidated to understand the molecular changes, making studying these changes increasingly relevant [4]. At the molecular level, CRSwNP is associated with Th2 cytokine polarization and an elevated type-2 inflammatory response [5], whereas CRSsNP is linked to Th1-dominant inflammatory response. Key cytokines, including IL-4, IL-5, and IL-13, have been identified as significant contributors to the inflammatory response and the development of CRSwNP [6]. The worsening of CRSwNP is attributed to various factors, including dysregulation of both innate and adaptive immune responses, which increases susceptibility to bacterial infections [7,8,9]. Epithelium-derived bioactive substances, such as cytokines, exosomes, and complements, modulate both innate and adaptive immune responses, playing a crucial role in the development and progression of this disease [10]. Another factor that is important in studying the molecular changes in CRSwNP is its heterogeneity, which influences not only the clinical phenotype and its presentation but also the response to different treatments.

Normal sinus secretions are derived from extravasated plasma and serous cell proteins and contain albumin, making up about 15% of total proteins, glandular mucous cell acidic mucins, and glandular serous cell antimicrobial proteins like secretory immunoglobulin A (IgA, 15% of total protein), lysozyme (15%), and lactoferrin (4%). These secretions humidify, heat or cool, and clean inhaled air, while immune proteins provide a primary defense against infections [11]. In chronic sinusitis (symptoms lasting over 12 weeks), glandular hyperplasia occurs in the affected sinuses and nearby nasal mucosa, which significantly changes the composition and properties of the mucus. Additionally, inflammatory cell products may be present in this complex mixture [11]. Nasal lavage fluids (NLFs) have shown promise as alternative sources for biomarker discovery. Numerous studies have shown that NLFs can effectively monitor changes caused by airway diseases such as asthma [12], acute sinusitis [11], and seasonal allergic rhinitis [13]. These findings support the use of NLFs to identify biomarkers in various diseases.

Proteomics offers a powerful approach to investigate dynamic changes within the proteome between two different conditions. The significant differentially expressed proteins can then serve as the biomarkers of disease. Previous studies have utilized 1D gel electrophoresis followed by LC-MS/MS analysis of nasal NLFs [14,15]. Another group conducted serum proteomic analysis [16] using capillary liquid chromatography–electrospray–quadrupole-time-of-flight mass spectrometry [17]. However, there has been limited research on NLF proteomics in patients with CRSwNP, particularly regarding its relationship with disease recurrence, which remains poorly understood.

To address this knowledge gap, we employed the LC-MS/MS label-free quantitative bottoms-up approach with data-dependent analysis to conduct NLF proteomic analysis to explore potential proteins associated with CRSwNP. Further insight into the pathogenesis of CRSwNP is critical for its management and diagnosis. Better identification might permit individualization of therapy with the potential for more effective treatment and better patient outcomes [1,18]. In this study, we aim to compare the proteomic analysis of NLFs between individuals diagnosed with CRSwNP and controls to gain a better understanding of the changes in the proteome and the associated biomarkers.

## 2. Materials and Methods

### 2.1. Ethical Considerations and Informed Consent

The study protocol and procedures used in the study were approved by the Institutional Review Board, College of Medicine, King Saud University (no. E- 23-7826)) prior to undertaking the study. All the participants provided written informed consent. The study was performed in accordance with the ethical standards of the Declaration of Helsinki and the universal International Conference on Harmonization Good Clinical Practice Guidelines.

### 2.2. Study Design and Subjects

A prospective cohort study of patients and control participants was conducted at the outpatient clinics of the Otorhinolaryngology Department, KAUH hospital, College of Medicine, King Saud University. A group of 18-year-old and older patients was recruited for the study. A total of 20 patients were recruited for the study. The inclusion criteria of the study included 10 patients diagnosed with CRSwNP according to EPOS2020 criteria [1,18]. This included new cases of CRSwNP, as well as those who had previous surgeries but presented with recurrent nasal polyposis on endoscopy and had been off medication for at least 6 weeks. Additionally, there were 10 healthy controls who attended the clinic for consultations without any nasal complaints.

The study excluded patients with autoimmune diseases or nasal tumors; individuals who had been prescribed specific medications such as oral or intranasal corticosteroids for 4–6 weeks; or any usage of biologics, leukotrienes, or muscarinic receptor antagonists within the previous 6 months. Clinical chemistry and anthropometric measures included the SNOT-22 (Sino-nasal Outcome Test 22), olfactory visual analog scale (VAS) score, total Nasal Polyp Score (NPS), and Lund–Mackay Score.

### 2.3. Sample Collection

Nasal lavage fluid samples were collected from the subjects. The nasal lavage fluid samples were collected and stored at −80 °C until analysis in the Proteomics Resource Unit, Obesity Research Center, College of Medicine, King Saud University.

### 2.4. Sample Preparation for Proteomics

#### 2.4.1. Nasal Lavage Fluid (NLF) Sample Preparation and Protein Extraction

The protein extraction from nasal lavage fluid (NLF) samples (40 mL) was performed by a modified protocol which was published earlier [19]. Briefly, NLF samples were centrifuged at 3000 rpm for 20 min at 4 °C, the supernatant was removed, and the pellet was added with 2-fold prepared lysis buffer (pH 8.8, 30 mM Tris-HCl, 7 M urea, 2 M thiourea, 2% CHAPS, and 1 × protease inhibitor mix). The samples were vortexed for 2 min and were shaken for 1 h at 4 °C. Then, they were sonicated (Microsonicator, Qsonica Sonicators, Newtown, CT, USA; 30% pulse, three intervals of 1 min each, separated by a 1 min gap). The samples were centrifuged at 15,000 rpm for 15 min. Then, 4× ice-cold acetone was added to the supernatant and kept at −20 °C overnight. The next day, the samples were centrifuged at 15,000 rpm for 15 min and the supernatant was discarded; the remaining protein pellets were dissolved in 2-fold lysis buffer. The protein concentration of each sample was determined in triplicate using the 2D-Quantkit (GE Healthcare, Piscataway, NJ, USA).

#### 2.4.2. Protein Quantification and Digestion for Insolution Digestion

Fifty micrograms of nasal lavage fluid proteins were transferred to a tube containing 10 µL of urea denaturing buffer (6 M urea). Disulfide bonds from the NLF proteins were reduced by adding 1 µL of dithiothreitol (200 mM) and incubated for 30 min at 60 °C. Afterward, the samples were alkylated by adding 1 µL of iodoacetamide (400 mM) solution and incubated at room temperature for another 30 min in the dark. The samples were diluted with 65 µL of ammonium bicarbonate buffer (50 mM) and digested overnight at 37 °C by adding 2.5 µL of sequencing-grade-modified trypsin (Promega, Madison, WI, USA) (1 µg/μL). To acidify the samples, 7 µL of 10% formic acid was added, and subsequently, the samples were desalted using Pierce C18 spin columns (Thermo Scientific, Waltham, MA, USA), and peptide concentration was determined using a Pierce Quantitative Colorimetric Peptide Assay (Thermo Scientific™ Pierce) [20,21]. The peptides eluted from the Pierce C18 spin were dried using vacuum centrifugation (Eppendorf Concentrator plus TM, Eppendorf, Germany).

#### 2.4.3. Liquid Chromatography Coupled to Tandem Mass Spectrometry (LC-MS/MS)

Peptides were reconstituted in a solution containing 0.1% (*v*/*v*) formic acid, and then, 1 µL of each sample was applied to a Dionex UltiMate 3000 nano-LC system with a WPS-3000 autosampler. The peptide was injected and concentrated on a PepMap100 C18 trap column (3 µm, 100 Å, 75 µm 285 inner diameter [i.d.] × 20mm, nanoViper; Thermo Scientific) that was equilibrated with 0.05% trifluroacetic acid in water. The selective trapping nano-LC setting achieved high-capacity sample loading. After switching the trap column inline, LC separations were performed with an analytical column (PepMap™ C18, 50 cm × 75 μm). Peptide separation was carried out at 300 nL/min with mobile phase A, which consisted of 0.1% (*v*/*v*) formic acid in water, while mobile phase B contained 0.1% (*v*/*v*) formic acid and 80% (*v*/*v*) acetonitrile in water. The column was pre-equilibrated with 5% mobile phase B, followed by an increase to 22.5% mobile phase B over 139 min, then 45% mobile phase B over 184 min. After separation, the peptides were injected into the nanospray ion source for ionization and analyzed by a Q Exactive Plus Hybrid Quadrupole-Orbitrap mass spectrometer (Thermo Fisher Scientific, Waltham, MA, USA) operating in positive ion mode, with nano electrospray (nESI) potential at 2000 V with a maximal duty cycle of 3 s. The scanning range for primary mass spectrometry (MS) was set to 375–1650 *m*/*z*, and the scanning resolution was set to 70,000; the fixed starting point of the scanning range for secondary mass spectrometry (MS/MS) was set to 80 *m*/*z*, and the scanning resolution for MS/MS spectrometry was set to 17,500. The dynamic exclusion time was set to 20 s, and the automatic gain control was set to 3 × 106 and 1 × 105 for MS and MS/MS scans, respectively. Mass spectra were acquired in a data-dependent mode (DDA).

### 2.5. Data Processing

MS and MS/MS raw data were analyzed using Proteome Discoverer v3.0 (Thermo Fisher Scientific, Bremen, Germany), with Sequest as the search engine and HUMAN-refprot-isoforms.fasta as the sequence database. Parameters applied for the analysis were the following: The search parameters included (1) pre-cursor/fragment mass tolerance: 15 ppm/0.02 Da; (2) maximal missed cleavages: 2; (3) enzyme name: trypsin (full); (4) dynamic modifications: peptide N-terminal acetylation, methionine oxidation; and (5) static modification: cysteine carbamidomethylation. Protein filtering was conducted with the protein FDRs being set at 1%, with a minimum of two unique peptides per protein. The filtered protein list was exported and manually formatted in Excel. Multivariate statistical analysis was evaluated using MetaboAnalyst v. 6.0 (McGill University, Montreal, QC, Canada) (http://www.metaboanalyst.ca, accessed on 12 March 2024) [22]. Only proteins that were identified and quantified with LFQ intensity were used for downstream analysis.

### 2.6. Statistical and Bioinformatics Analysis

All statistical comparisons between groups were performed using Student’s t-test implemented by Proteome Discoverer v3.0. Adjusted *p*-values after FDR (q-values) were considered significant for values below 0.01. The differently expressed significant proteins (FDR *p*-value ≤ 0.05-fold change ≥1.5) were exported from the Proteome Discoverer. Ingenuity pathway analysis (IPA) was carried out by importing quantitative data into the IPA software (Ingenuity Systems, http://www.ingenuity.com) version Q2 2024 (https://digitalinsights.qiagen.com/IPA, accessed on 4 July 2024) (Qiagen, Aarhus, Denmark). This software aids in determining the functions and pathways that are most strongly associated with the protein list by overlaying the experimental expression data on networks constructed from published interactions. Furthermore, the PANTHER (protein analysis through evolutionary relationships) classification system (http://www.pantherdb.org, accessed on 20 July 2024) was used to categorize the identified proteins based on their molecular function and biological process. This section may be divided by subheadings. It should provide a concise and precise description of the experimental results, their interpretation, and the experimental conclusions that can be drawn.

## 3. Results

### 3.1. Clinical and Biochemical Characteristics of Study Subjects

Participants’ demographics and clinical profiles are summarized in Table 1. The CRSwNP group (n = 10) had a mean age of 41.7±7.9 years, while the control group (n = 10) had a mean age of 35.7 ± 7.2 years. In the CRSwNP group, there were six males and four females, whereas in the control group, there were four males and six females. Smoking status varied among participants, with only two smokers in the CRSwNP group, compared to one smoker and three previous smokers in the control group. Notably, eight participants in the CRSwNP group had undergone previous sinonasal surgery, while none of the control had a history of such surgery. Allergic rhinitis was prevalent among the CRSwNP group (n = 10) but less common among control (n = 2). Additionally, five CRSwNP and one control reported a history of asthma. Clinical assessment was done using patient-reported outcome measures for chronic rhinosinusitis with the Sino-nasal Outcome Test (SNOT-22). The scores differed between groups, with the CRSwNP group exhibiting higher SNOT-22 scores (mean 38.2 ± 8.5) compared to the control group (mean 6.8 ± 9.6). Moreover, the CRSwNP had a lower olfactory VAS score (mean 3.6 ± 3.7) than the control (mean 9.7 ± 0.9). The total nasal polyp score and Lund–Mackay scores in the CRSwNP group had mean scores of 3.8 ± 1.6 and 19 ± 5.5, respectively.

### 3.2. Proteomic Analysis and Identification of Differentially Expressed Proteins

#### 3.2.1. Label-Free Quantitative Proteomics Analysis

Label-free quantitative proteomics was used to compare samples from the two groups (control and CRSwNP). In total, 2768 non-redundant proteins were quantified based on identifying one or more unique peptides. Significant and differentially expressed proteins were defined as those that showed a fold change greater than 1.5 or less than 0.66 in relative abundance and a *p*-value < 0.05. Based on these criteria, we identified 234 proteins (151 up- and 83 down-regulated) as significantly differentially regulated between the control and CRSwNP groups (Supplementary Material S1). 

#### 3.2.2. Analysis of Differentially Expressed Proteins in CRSwNP

**Figure 1:**
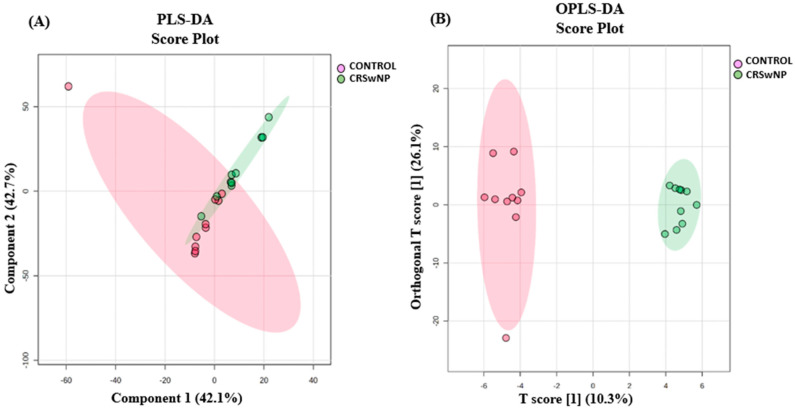
Multivarate analysis of proteomic profiles of patients with CRSwNP and control groups. (**A**) Partial least squares discriminant analysis (PLS-DA) displays semi-separation between the CRSwNP and control groups. (**B**) OPLS-DA shows a clear separation between the two groups, indicating a significant proteomic difference between the control and CRSwNP groups. The robustness of the created models was evaluated by the fitness of the model (R2Y = 0.987) and predictive ability (Q2 = 0.657) values in a larger dataset (n = 100).

**Figure 2:**
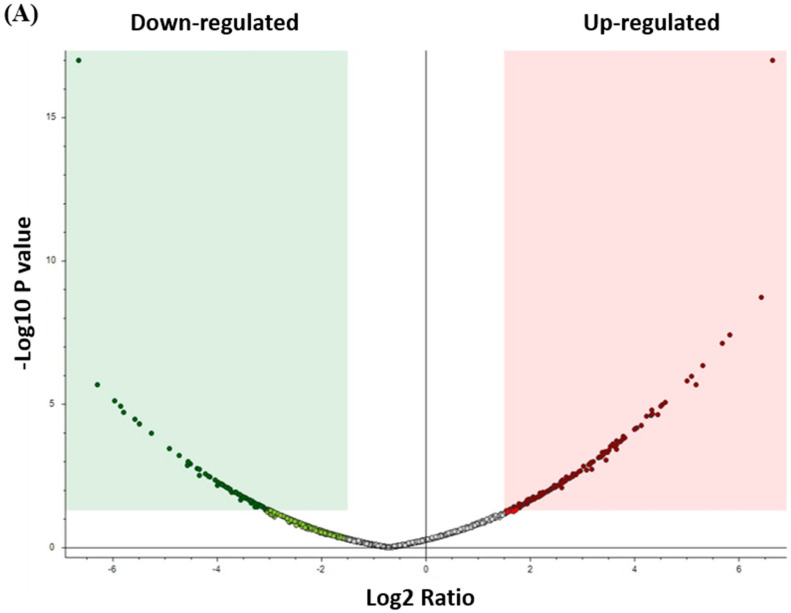

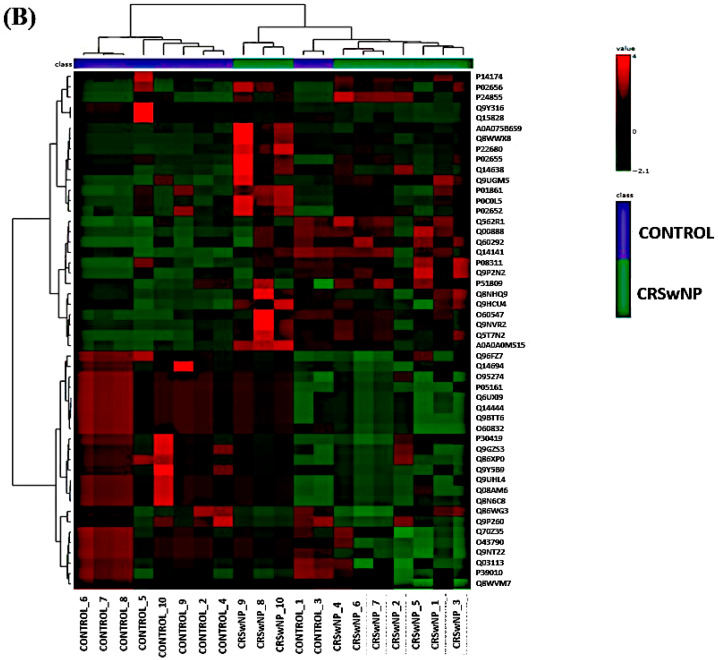
(**A**) The volcano plot shows a significant change in the levels of several proteins, of which green represents down-regulated and red represents up-regulated proteins in the control vs. CRSwNP groups (FDR *p*-value ≤ 0.05-fold change ≥1.5). (**B**) Hierarchal Clustering (HAC) and heat map analysis of identified proteins that significantly altered between the control and CRSwNP groups. The color range bar indicates down-regulated proteins as green and up-regulated proteins as red.

The proteins that distinguished between the control and CRSwNP group are displayed in Figure 1. The visualization of each study group and outlier detection was carried out using partial least squares discriminant analysis (PLS-DA) (Figure 1A), and orthogonal partial least squares discriminant analysis (OPLS-DA), a supervised multivariate approach, was applied and displayed an OPLS-DA model score plot (Figure 1B). We utilized OPLS-DA for training and classification to distinguish between the two groups. OPLS-DA was chosen for its ability to separate predictive variance from orthogonal variance, enhancing both the interpretability and performance of the model. Key parameters optimized during the analysis included the number of latent variables and orthogonal components, along with appropriate scaling to prevent overfitting. Given the small sample size of 20 samples divided into two groups, we applied k-fold cross-validation (5-fold) to ensure the robustness of the model. In each fold, the data were split into 80% for training and 20% for validation, with all samples participating in both training and validation at least once. Although the limited sample size may impact the model’s generalizability, cross-validation helped mitigate bias. Expanding the sample size in future studies will be essential to further validate the findings and improve the model’s reliability. The distinct separation of the control and CRSwNP groups suggests that proteins may be a useful tool for identifying the effect (CRSwNP). The robustness of the created models was evaluated by the fitness of the model (R2Y = 0.987) and predictive ability (Q2 = 0.657) values in a larger dataset (n = 100). A moderate t-test (*p*-value < 0.05) and fold change (FC cutoff of 1.5) were used to analyze the volcano plot between the control and CRSwNP groups. The results showed that from a total of 234 dysregulated proteins, 83 (green) and 151 (red) proteins were down-regulated and up-regulated in the control vs. CRSwNP groups, respectively (Figure 2A). The proteins with a notable difference between the control and CRSwNP groups are well represented by the heat map (Figure 2B). Thus, they might be considered possible protein biomarkers to identify the molecular, biological, and cellular changes that occur in CRSwNP. The most dysregulated proteins between the control and CRSwNP groups are shown in Table 2.

#### 3.2.3. Evaluation of Protein Biomarkers Between Study Groups

**Figure 3:**
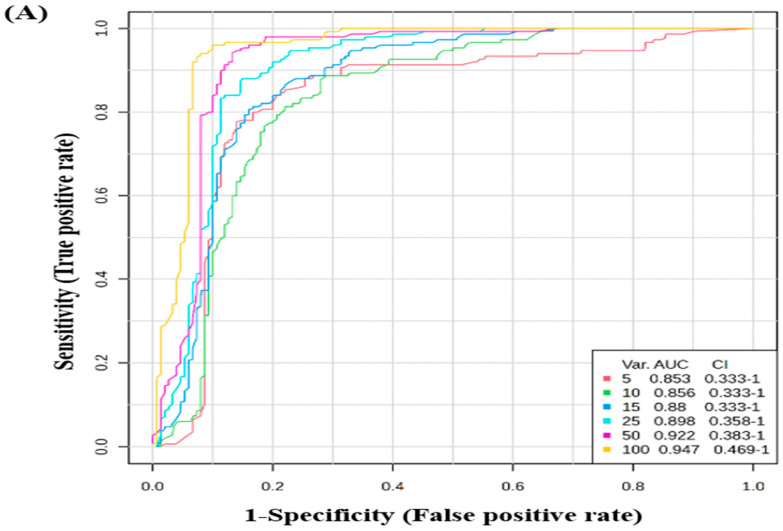

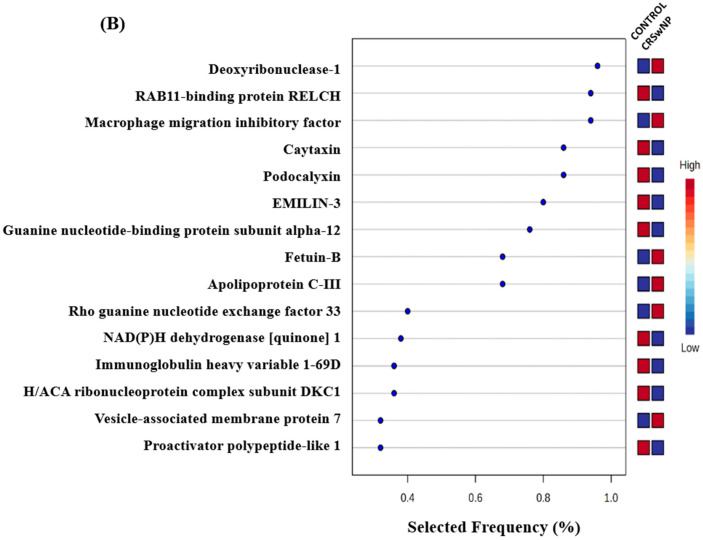
Biomarker evaluation in control vs. CRSwNP. (**A**) The Receiver Operating Characteristic (ROC) curve was generated by the OPLS-DA model, with Area Under the Curve (AUC) values calculated from the combination of 5, 10, 15, 25, 50, and 100 proteins. (**B**) Frequency plot showing the top 15 significantly dysregulated identified protein biomarkers in the control vs. CRSwNP groups.

The potential biomarkers were evaluated using the Receiver Operating Characteristic (ROC) curve. PLS-DA was used as a classification and feature ranking approach to create a multivariate exploratory ROC analysis. Ten features at the exploratory ROC curve using PLS-DA with cross-validation (CV) had an Area Under the Curve (AUC) value of at least 0.947 (95% CI) (Figure 3A). The frequency plot of the top 15 significantly dysregulated identified protein biomarkers in the control vs. CRSwNP groups showed Deoxyribonuclease-1, Macrophage migration inhibitory factor, Fetuin-B, Apolipoprotein C-III, Rho guanine nucleotide exchange factor 33, and Proactivator polypeptide-like 1 were up-regulated in the CRSwNP group, whereas RAB11-binding protein RELCH, Caytaxin, Podocalyxin, EMILIN-3, Guanine nucleotide-binding protein subunit alpha-12, NAD(P)H dehydrogenase [quinone] 1, Immunoglobulin heavy variable 1–69 D, H/ACA ribonucleoprotein complex subunit DKC1, and Proactivator polypeptide-like 1 were down-regulated in the CRSwNP group in comparison to the control group (Figure 3B).

#### 3.2.4. Evaluation of the Top Four Protein Biomarkers Between the CRSwNP and Control Groups

**Figure 4:**
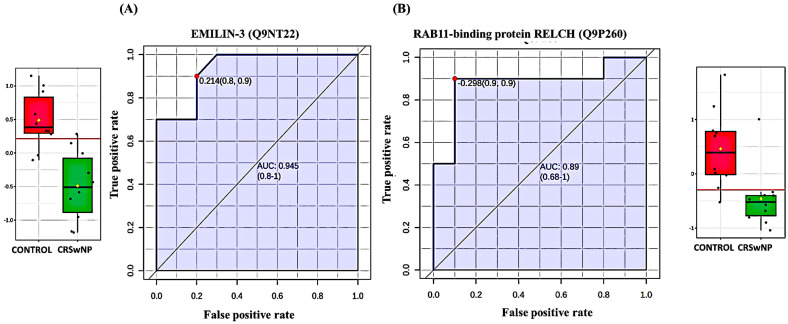
Two down-regulated proteins in CRSwNP in comparison with a control group with the highest AUC. (**A**) EMILIN-3 (Q9NT22), AUC = 0.945; box plot (FDR *p* ≤ 0.05 and fold change ≥1.5), where red represents the control and green represents the CRSwNP group. (**B**) RAB11-binding protein RELCH (Q9P260), AUC = 0.89; box plot (FDR *p* ≤ 0.05 and fold change ≥1.5), where red represents the control and green represents the CRSwNP group.

**Figure 5:**
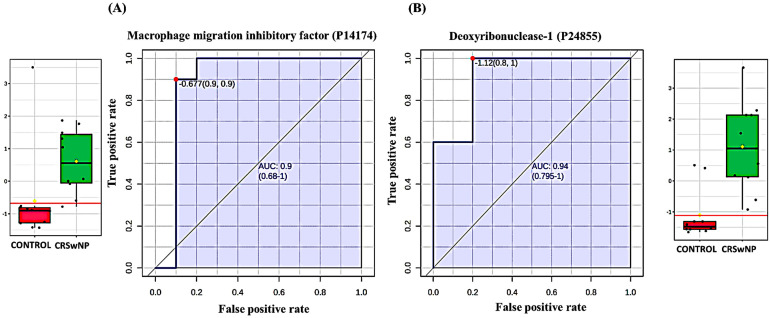
Two up-regulated proteins in the CRSwNP vs. control group with the highest AUC. (**A**) Macrophage migration inhibitory factor (P14174), AUC = 0.9; box plot (FDR *p* ≤ 0.05 and fold change ≥1.5), where red represents the control and green represents the CRSwNP group. (**B**) Deoxyribonuclease-1 (P24855), AUC = 0.94; box plot (FDR *p* ≤ 0.05 and fold change ≥1.5), where red represents the control and green represents the CRSwNP group.

The ROC curve’s AUC value for EMILIN-3 (Q9NT22) was 0.945 (Figure 4A) and its value for RAB11-binding protein RELCH (Q9P260) (Figure 4B) was 0.890, and both were down-regulated in the CRSwNP group. The box-and-whisker plots indicated decreased expression of EMILIN-3 and RAB11-binding protein RELCH in the CRSwNP group compared to the control group. The ROC curve’s AUC values for Macrophage migration inhibitory factor (P14174) (Figure 5A) and Deoxyribonuclease-1 (P24855) (Figure 5B) were 0.900 and 0.940, respectively, and were up-regulated in CRSwNP. The box-and-whisker plots indicated increased expression of Macrophage migration inhibitory factor and Deoxyribonuclease-1 in the CRSwNP group in comparison with the control group.

#### 3.2.5. Interaction Network Analysis of Differentially Expressed Proteins

**Figure 6:**
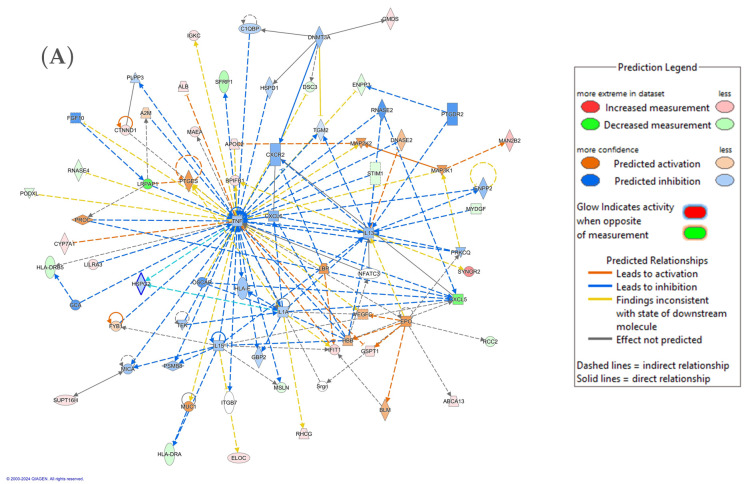

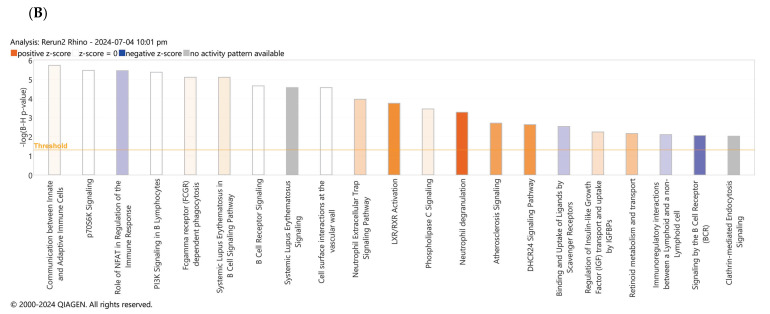
Schematic representation of the highest-scoring network pathways depicting the involvement of the differentially regulated proteins. Nodes colored blue represent down-regulation, and those colored orange represent up-regulation. (**A**) Protein interaction network pathway between the CRSwNP and control groups. (**B**) Top canonical pathways ranked by the *p*-values obtained by the IPA. Blue bars represent negative z-score, orange bars represent positive z-score, and grey bars represent no activity pattern available. The interaction networks were generated through IPA (QIAGEN Inc., Fresno, CA, USA).

The significantly differentially regulated proteins in our data were uploaded to the IPA software to determine the biological role of the proteins. The highest interactions for the proteins were related to cellular movement, inflammatory response, organismal injury and abnormalities pathway. This network included 30 proteins and had the highest score of 37. The proteins in the network were involved in regulating the TNF, IL-1, and IL-13 signaling pathways. The top canonical pathways included LXR/RXR activation, complement cascade, cell surface interactions at the vascular wall, DHCR24 signaling pathway, neutrophil extracellular trap signalling pathway, etc. (Figure 6A,B; Supplementary Material S2).

**Figure 7:**
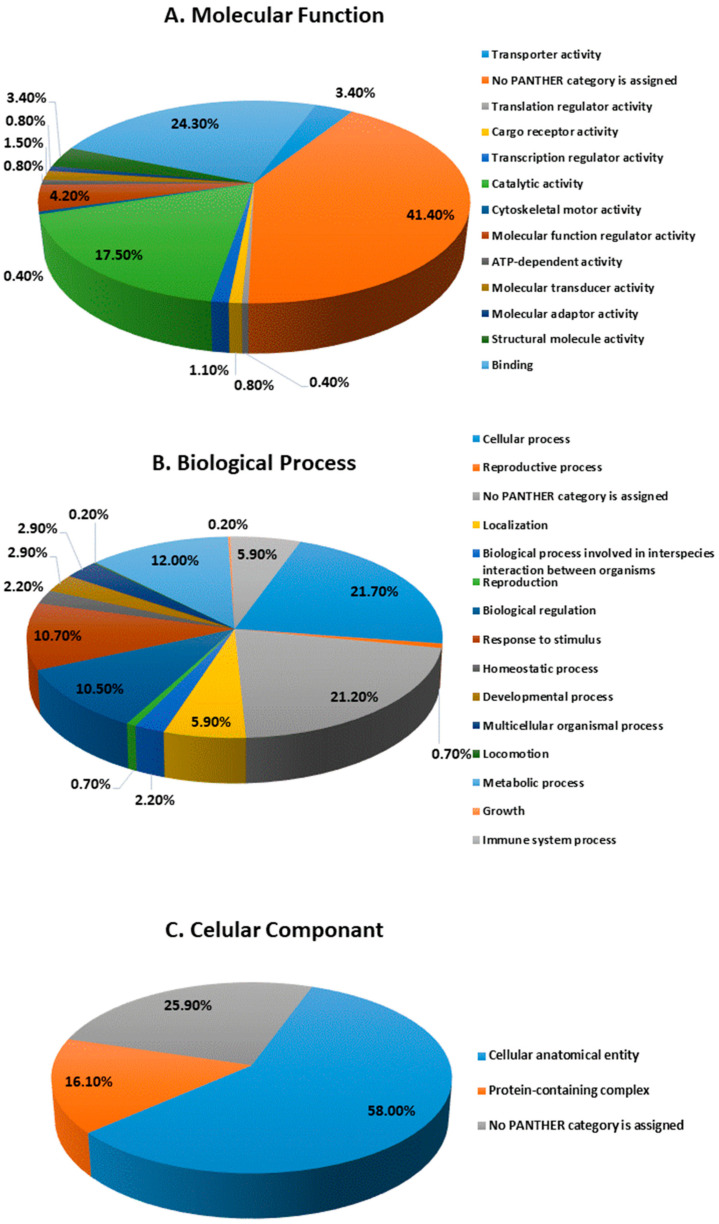
Comparative depiction of identified proteins categorized into groups according to their molecular function (**A**), biological process (**B**), and cellular components (**C**).

The protein analysis through evolutionary relationships (PANTHER) classification system was used to identify the proteins by their molecular functions (Figure 7A), biological processes (Figure 7B), and cellular components (Figure 7C). The functional category showed that most of the differentially expressed proteins identified were enzymes with binding (24.3%), followed by catalytic activity (17.5%) (Figure 7A). With regard to biological processes, the identified proteins were involved in cellular processes (21.70%) and metabolic processes (12.0%) (Figure 7B). The majority of the identified proteins were located in the cellular anatomical entity (58.0%), followed by the protein containing complex (16.1%) (Figure 7C).

## 4. Discussion

Our untargeted label-free quantitative LC-MS/MS analysis of NLFs between individuals diagnosed with CRSwNP and controls determined the significant dysregulations of proteins between the two groups. Currently, the etiology of CRSwNP has not been fully elucidated. Previous reports have indicated that epithelial cells, along with immune and inflammatory cells such as macrophages, T and B lymphocytes, eosinophils, neutrophils, and mast cells, play a significant role in disease pathology [23]. The underlying cause of CRS is considered to be systemic immune mucosal inflammation. The identified proteins are involved in the regulation of various metabolic pathways, including those related to oxidative stress and immune system regulation.

### 4.1. Role of Oxidative Stress in CRSwNP

In the present study, we identified oxidative stress, an imbalance between reactive oxygen species production and antioxidative defense activity, which is believed to have a role in the development and pathogenesis of nasal polyps and in the development of CRSwNP by impairment of the nasal epithelial barrier [8]. Excess ROS can be eliminated through the action of various enzymes that act as antioxidants, including superoxide dismutase (SOD), GSTM3, catalase, and other components [24]. In our proteomic analysis, we identified a decrease in the levels of SOD, GSTM3, and GGT7 in patients with CRSwNP. The decrease in these enzymes points to an increase in the levels of oxidative stress in this group. Previous studies have also shown that oxidative stress plays an important role in the development of nasal polyps and CRSwNP. The increase in the levels of oxidative stress was also found to be positively correlated with the cytokine levels [25,26,27]. In a study by Zhou et al., the expression levels of NOS2, NOX1, HO-1, and SOD2 were found to be increased in nasal epithelial cells and macrophages derived from nasal polyp tissue [25].

### 4.2. Role of Immune Cell Dysregulation and Inflammation in CRSwNP

In this study, we identified seven proteins forming a protein cluster involved in immune cell dysregulation, especially in the degranulation of neutrophils and regulation of the neutrophil extracellular trap signaling pathway. These included ELANE, GSDMD, LTF, MIF, DNASE1, CTSG, and DPP7. The differences in the immune profile based on the cytokines involved in the body’s innate and adaptive immune response are generally used to differentiate between the different endotypic subtypes of CRS. Previous studies have shown that a T-helper 2 (Th2) cytokine-predominant immune profile has been linked to CRSwNP [28,29] and characterized as both eosinophilic and non-eosinophilic subtypes [18], with a strong dependence on geographical regions. The eosinophilic CRSwNP subtypes have been predominantly observed in European and American populations, whereas non-eosinophilic CRSwNP was considered the most common form in Japan [30,31,32,33]. Additionally, recent studies from China have shown a predominance of neutrophilic CRSwNP subtypes with mixed Th1/Th17 inflammatory profiles, which are observed prominently in Asian CRSwNP patients [34,35,36]. A previous study of patients with CRSwNP subtypes in Saudi Arabia also exhibited a Th2 inflammatory pattern, with diffuse tissue neutrophil infiltration observed in about 60% of cases and focal infiltration in another 15% [37]. Neutrophils are predominant innate immune cells that play an essential role in protecting the body against infection through various mechanisms, including phagocytosis, degranulation, and the formation of neutrophil extracellular traps (NETs). NETs, in turn, are extracellular structures released by activated neutrophils, consisting of granule proteins including ELANE, GSDMD, LTF, MIF, DNASE1, CTSG, DPP7, NADPH oxidase, and extracellular DNA [38]. In this regard, our proteomic analysis identified neutrophil elastase, cathepsin G. In a recent study, both eosinophilic and neutrophilic extracellular traps were identified to play a significant role in chronic rhinosinusitis [39]. In addition, we found a decrease in the levels of the glycoprotein EMILIN, which is known to be reduced by the action of ELANE to promote binding and cell proliferation [40]. We also noted an increase in Macrophage migration inhibitory factor (MIF) and multi-effect proinflammatory factor, expressed in monocytes, macrophages, eosinophils, neutrophils, B cells, and T cells [9,10]. MIF promotes the inflammatory response, eosinophil differentiation, activation, migration, and survival. Our findings align with those of Yuan et al., who also showed increased MIF in patients with CRSwNP [41]. Our proteomic analysis also revealed an increase in levels of the enzyme DNASE1. The increase in the levels of this enzyme also points to the increased inflammatory action of the neutrophils through the formation of NETs and breakdown products after neutrophil action [42]. Although our population of CRSwNP patients predominantly belongs to the Th2 inflammatory pattern, as mentioned earlier, neutrophilic innate immunity regulation might also play a significant role, particularly in the recurrence of nasal polyposis, as shown in this study.

Interestingly, our study identified an increase in IL-36 in the CRSwNP proteomics data. This finding aligns with previous studies where IL-36γ was the most detected isoform in CRS mucosa [43,44]. The IL-36 cytokine family (IL-36α, IL-36β, and IL-36γ) is believed to play an important role in regulating adaptive immune responses in the sinonasal mucosa in both CRS without nasal polyps (CRSsNP) and CRSwNP. This process occurs after Toll-like receptor (TLR) activation by various stimuli, such as microbial elements, leading to the subsequent release of a complex cascade of chemokines that disrupt the integrity of endothelial junctions [43].

### 4.3. Biomarkers of Chronic Rhinosinusitis with Polyposis and Network Pathway Analysis

Our present study has provided proteomics analysis with bioinformatics and network pathway analysis for CRSwNP. We identified significantly different proteins between patients with chronic rhinosinusitis with polyps and normal individuals. The top proteins identified in the biomarker analyses related to the role of neutrophils in CRSwNP, including deoxyribonuclease 1, MIF, EMILIN 3, and NADPH dehydrogenase. Additionally, we performed network pathway analysis of the significantly differentially regulated proteins. The interacting proteins highlighted key pathways related to cellular movement, inflammatory response, organismal injury, and abnormalities. The top network pathway had a score of 37, with 35 interacting proteins. The majority of the proteins were involved in regulating the TNF signaling pathway along with the regulation of the cytokines that included interleukin-1α (IL-1α) and interleukin-13 (IL-13). IL-1α is an immunomodulating protein secreted by stimulated macrophages, monocytes, natural killer cells, fibroblasts, and epithelial cells, along with IL-1β. Both IL-1α and IL-1β share a similar receptor, and together, they stimulate and proliferate the inflammatory pathway [45]. Moreover, our network protein analysis revealed an abundance of proteins involved in cytokine pathways, including IL-13, considered a crucial cytokine in regulating immune homeostasis, with diverse functions such as the activation of various inflammatory cells, transformation of monocytes into macrophages, and stimulation of eosinophils, also it induces goblet cell hyperplasia and rapid overgrowth of smooth muscle cells, and increases the permeability of the sinonasal mucosal barrier [46,47,48]. The involvement of IL-1 and IL-13 points to a probable mixed cytokine (TH1 and TH2) response in our group of patients.

Limitations include that this prospective cohort study employs an untargeted, label-free LC-MS/MS approach to analyze proteomic profiles in nasal lavage fluid from patients with chronic rhinosinusitis with nasal polyposis (CRSwNP) and healthy controls. Bioinformatics and network pathway analysis identified potential biomarkers, supported by a high Area Under the Curve (AUC) value of 0.999, demonstrating their clinical relevance. Although focused on a specific cohort, the findings may be applicable to similar populations. The small sample size (n = 10 per group) limits the generalizability of the results, and the characteristics of the cohort may affect broader applicability. The lack of longitudinal follow-up prevents an assessment of biomarker stability and predictive value over time. Given the low number of smokers in both groups, smoking likely had minimal impact on the outcomes, though it may act as a confounding factor in CRSwNP progression. Further investigation in larger cohorts is warranted. Additionally, the focus on proteomics alone limits insights into the underlying disease mechanisms. Future research with larger cohorts and longer follow-up is needed to validate these findings.

## 5. Conclusions

Our study has provided functional insights into the proteomic changes between CRSwNP and individuals without sinusitis. The significantly dysregulated proteins identified in this group showed the presence of heightened markers of inflammation and oxidative stress that can be considered pathophysiological for developing this disease. More mechanistic studies using larger cohorts are needed to ascertain the utility of the identified biomarkers.

## Figures and Tables

**Table 1:** Participants’ demographics and clinical profiles.

		CRSwNP (n = 10)	Control (Without CRS) (n = 10)
Age (Mean ± SD)		41.7 ± 7.9	35.7 ± 7.2
Gender	Male	6	4
	Female	4	6
Smoking Status *	Smoker	2	1
	Non-smoker	8	6
	Previous smoker	0	3
Previous Sinonasal Surgery	Yes	8	0
	No	2	10
Allergic Rhinitis	Yes	10	2
	No	0	8
Asthma	Yes	5	1
	No	5	9
SNOT-22 Score (Mean ± SD)		38.2 ± 8.5	6.8 ± 9.6
Total Nasal Polyp Score ** (Mean ± SD)		3.8 ± 1.6	0
Lund–Mackay Score (Mean ± SD)		19 ± 5.5	2
Olfactory VAS Score (Mean ± SD)		6.4 ± 3.7	0.3 ± 0.9

CRSwNP, chronic sinusitis with nasal polyps; CRS chronic rhinosinusitis; SD, standard deviation; SNOT-22, Sino-nasal Outcome Test 22. * This encompasses traditional cigarettes, traditional shisha, and electronic shisha. ** Grading based on the Lidholt Polyp Grading Score System.

**Table 2:** Top 10 dysregutated proteins between control and CRSwNP groups.

	Accession	Description	Coverage [%]	Abundance Ratio: (CRSwNP)/(CONTROL) Calculated	Up-Regulated/ Down-Regulated	Abundance Ratio (log2): (CRSwNP)/(CONTROL)	Abundance Ratio *p*-Value: (CRSwNP)/(CONTROL)	Abundance Ratio Adj. *p*-Value: (CRSwNP)/(CONTROL)
1	Q6NUJ1	Proactivator polypeptide-like 1	2	−20.00	DOWN	−4.33	0.00307836	0.0351557
2	Q8TD33	Secretoglobin family 1C member 1	40	−18.52	DOWN	−4.22	0.00276391	0.0319461
3	Q9NX62	Golgi-resident adenosine 3',5'-bisphosphate 3'-phosphatase	9	−17.86	DOWN	−4.16	0.00324536	0.0364035
4	Q01581	Hydroxymethylglutaryl-CoA synthase, cytoplasmic	3	−17.54	DOWN	−4.13	0.00351064	0.0391009
5	Q14141	Septin-6	6	−16.39	DOWN	−4.03	0.00455383	0.0489886
6	A8MVX0	Rho guanine nucleotide exchange factor 33	3	16.59	UP	4.05	6.7006 × 10^−5^	0.001001
7	Q9Y2E5	Epididymis-specific alpha-mannosidase	2	17.51	UP	4.13	5.5722 × 10^−5^	0.0008364
8	P51649	Succinate-semialdehyde dehydrogenase, mitochondrial	6	18.77	UP	4.23	2.6305 × 10^−5^	0.0004045
9	Q71DI3	Histone H3.2	66	19.95	UP	4.32	2.5421 × 10^−5^	0.0003928
10	Q08AM6	Protein VAC14 homolog	3	20.02	UP	4.32	2.4953 × 10^−5^	0.0003875

## Data Availability

The datasets presented in this study can be found in the online MassIVE repository via accession ID: MSV000096171.

## Supplementary Materials

The following supporting information can be downloaded at: https://www.mdpi.com/article/10.3390/biology13110887/s1, Supplementary Material S1: Details of the significantly identified proteins between the CRSwNP and control group; Supplementary Material S2: The biological impact of the changes in the abundance of proteins examined using IPA software.
