# Supplementary material for: Label-Free Quantitative Proteomics Analysis of Nasal Lavage Fluid in Chronic Rhinosinusitis with Nasal Polyposis
Source: Biology (Basel). 2024 Oct 30;13(11):887. doi: 10.3390/biology13110887 (PMC11591906; doi:10.3390/biology13110887)
Supplement: Supplementary file 1 [file biology-13-00887-s001.zip › Supplementary data2.pdf]

INGENUITY
PATHWAY ANALYSIS

Analysis Name: Rerun2 Rhino - 2024-07-04 10:01 pm
Analysis Creation Date: 2024-07-04
Build version: exported
Content version: 111725566 (Release Date: 2024-03-21)

Experiment Metadata

Name	Value
originalColumnNames	Accession,Description

Analysis Settings

Reference set: Ingenuity Knowledge Base (Genes Only)
Relationship to include: Direct and Indirect
Does not Include Endogenous Chemicals
Optional Analyses: My Pathways My List

Filter Summary:
Consider only molecules and/or relationships where
(species = Human OR Uncategorized OR Mouse OR Rat) AND
(confidence = Experimentally Observed) AND
(tissues = Effector memory helper T cells OR Peripheral blood monocytes OR Mast cells OR Peritoneal macrophages OR NK cells not otherwise specified OR Monocyte-derived macrophage OR Eosinophils OR Granulocytes not otherwise specified OR Other Granulocytes OR Monocytes not otherwise specified OR Effector memory cytotoxic T cells OR Myeloid dendritic cells OR Bone marrow-derived macrophages OR Naive

(c) 2000-2024 QIAGEN. All rights reserved.

helper T cells OR Peripheral blood lymphocytes OR Thymocytes OR Other Endothelial cells OR Endothelial cells not otherwise specified OR Other Bone marrow cells OR Blood platelets OR Other Monocytes OR Activated CD56dim NK cells OR Effector memory RA+ cytotoxic T cells OR Fibroblasts OR Activated helper T cells OR Activated Vd1 Gamma-delta T cells OR Activated CD56bright NK cells OR Immature monocyte-derived dendritic cells OR Immune cells not otherwise specified OR Plasma cells OR Other Memory T lymphocytes OR Other Monocyte-derived dendritic cells OR Skin OR Other NK cells OR Hematopoietic progenitor cells OR Bone marrow-derived dendritic cells OR CD4+ T-lymphocytes OR Bone marrow cells not otherwise specified OR Cells not otherwise specified OR Central memory cytotoxic T cells OR Intraepithelial T lymphocytes OR Macrophages not otherwise specified OR Skeletal Muscle OR Activated Vd2 Gamma-delta T cells OR Effector T cells OR Mature monocyte-derived dendritic cells OR Other Dendritic cells OR Memory T lymphocytes not otherwise specified OR CD34+ cells OR Microglia OR Th2 cells OR Astrocytes OR Hepatocytes OR BDCA-1+ dendritic cells OR Central memory helper T cells OR Plasmacytoid dendritic cells OR Mononuclear leukocytes not otherwise specified OR Other Peripheral blood leukocytes OR Other Macrophages OR BDCA-3+ dendritic cells OR Th1 cells OR Tissues and Primary Cells not otherwise specified OR Trachea OR Microvascular endothelial cells OR Other Organ Systems OR Murine NKT cells OR Langerhans cells OR Other Immune cells OR Melanocytes OR Memory B cells OR Dendritic cells not otherwise specified OR PBMCs OR Monocyte-derived dendritic cells not otherwise specified OR Megakaryocytes OR CD56bright NK cells OR Keratinocytes OR Cytotoxic T cells OR Adipocytes OR Natural T-regulatory cells OR Epithelial cells not otherwise specified OR Neutrophils OR Peripheral blood leukocytes not otherwise specified OR Lung OR Th17 cells OR CD56dim NK cells OR T lymphocytes not otherwise specified OR Lymphocytes not otherwise specified OR Other Mononuclear leukocytes) AND
(mol. types = canonical pathway OR chemical - endogenous mammalian OR complex OR cytokine OR disease OR enzyme OR function OR G-protein coupled receptor OR group OR growth factor OR ion channel OR kinase OR ligand-dependent nuclear receptor OR microRNA OR other OR peptidase OR phosphatase OR transcription regulator OR translation regulator OR transmembrane receptor OR transporter) AND
(data sources = An Open Access Database of Genome-wide Association Results OR BIND OR BioGRID OR Catalogue Of Somatic Mutations In Cancer (COSMIC) OR Chemical Carcinogenesis Research Information System (CCRIS) OR Clinical Genome Resource (ClinGen) OR ClinicalTrials.gov OR ClinVar OR Cognia OR DIP OR DrugBank OR Gene Ontology (GO) OR GVK Biosciences OR Hazardous Substances Data Bank (HSDB) OR HumanCyc OR Ingenuity Expert Findings OR Ingenuity ExpertAssist Findings OR IntAct OR Interactome studies OR MIPS OR miRBase OR miRecords OR Mouse Genome Database (MGD) OR Obesity Gene Map Database OR Online Mendelian Inheritance in Man (OMIM) OR Reactome OR TarBase OR TargetScan Human OR TargetScan Mouse)

Top Canonical Pathways

(c) 2000-2024 QIAGEN. All rights reserved.

Name	p-value	Overlap
**B Cell Development**	5.52E-12	10.1 % 19/189
**IL-15 Signaling**	1.54E-09	7.7 % 18/233
**FcRIIB Signaling in B Lymphocytes**	2.32E-09	7.5 % 18/239
**Communication between Innate and Adaptive Immune Cells**	1.19E-08	5.8 % 21/363
**Altered T Cell and B Cell Signaling in Rheumatoid Arthritis**	1.82E-08	5.6 % 21/372

Top Upstream Regulators

Upstream Regulators

Name	p-value	Predicted Activation
**taurocholic acid**	3.52E-04	
**UBE2I**	4.07E-04	
**HNF1A**	1.12E-03	
**PTPN11**	1.13E-03	
**FOS**	1.69E-03	

Causal Network

Name	p-value	Predicted Activation
**KITLG**	1.69E-07	

(c) 2000-2024 QIAGEN. All rights reserved.

**IL18**	2.51E-06
**ILK**	2.53E-06
**EDN1**	2.76E-06
**CD40**	2.94E-06

Top Diseases and Bio Functions

Diseases and Disorders

Name	p-value range	# Molecules
**Organismal Injury and Abnormalities**	4.99E-02 - 1.12E-05	66
**Inflammatory Response**	4.99E-02 - 1.19E-05	44
**Cardiovascular Disease**	3.83E-02 - 4.01E-05	10
**Hematological Disease**	4.42E-02 - 1.51E-03	15
**Immunological Disease**	4.42E-02 - 2.02E-03	18

Molecular and Cellular Functions

Name	p-value range	# Molecules
**Cellular Movement**	4.90E-02 - 1.03E-05	49
**Cell Morphology**	4.42E-02 - 4.01E-05	22
**Cellular Function and Maintenance**	4.31E-02 - 4.01E-05	50
**Cellular Assembly and Organization**	4.42E-02 - 4.16E-05	21
**Cell-To-Cell Signaling and Interaction**	4.61E-02 - 5.82E-04	38

(c) 2000-2024 QIAGEN. All rights reserved.

INGENUITY PATHWAY ANALYSIS

Physiological System Development and Function

Name	p-value range	# Molecules
Hematological System Development and Function	4.90E-02 - 1.03E-05	47
Immune Cell Trafficking	4.90E-02 - 1.03E-05	42
Cardiovascular System Development and Function	4.42E-02 - 4.01E-05	10
Tissue Morphology	4.82E-02 - 4.01E-05	14
Embryonic Development	3.92E-02 - 4.16E-05	8

Top Tox Functions

Assays: Clinical Chemistry and Hematology

Name	p-value range	# Molecules
Increased Levels of LDH	2.57E-02 - 2.57E-02	1
Increased Levels of Red Blood Cells	7.51E-02 - 7.51E-02	1

Hepatotoxicity

Name	p-value range	# Molecules
Liver Damage	1.57E-02 - 1.57E-02	2
Hepatocellular carcinoma	2.59E-01 - 3.83E-02	2

(c) 2000-2024 QIAGEN. All rights reserved.

INGENUITY PATHWAY ANALYSIS

**Liver Hyperplasia/Hyperproliferation**	2.59E-01 - 3.83E-02	2
**Glutathione Depletion In Liver**	6.30E-02 - 6.30E-02	1
**Liver Fibrosis**	6.30E-02 - 6.30E-02	1

Nephrotoxicity

Name	p-value range	# Molecules
**Glomerular Injury**	1.77E-02 - 1.77E-02	2
**Renal Fibrosis**	1.77E-02 - 1.77E-02	2
**Renal Hydronephrosis**	2.57E-02 - 2.57E-02	1

Top Regulator Effect Networks

Top Networks

ID	Associated Network Functions	Score
**1**	Cellular Movement, Inflammatory Response, Organismal Injury and Abnormalities	37
**2**	Cellular Movement, Immune Cell Trafficking, Cell Death and Survival	33

(c) 2000-2024 QIAGEN. All rights reserved.

3	Cellular Movement, Immune Cell Trafficking, Inflammatory Response	31
4	Cellular Movement, Cardiovascular System Development and Function, Organismal Development	28
5	Inflammatory Response, Organismal Injury and Abnormalities, Cell Death and Survival	28

Top Tox Lists

Name	p-value	Overlap
LXR/RXR Activation	4.58E-06	8.1 % 10/123
Renal Safety Biomarker Panel (PSTC)	2.42E-03	33.3 % 2/6
FXR/RXR Activation	2.60E-03	4.4 % 8/182
Negative Acute Phase Response Proteins	4.43E-03	25.0 % 2/8
Hepatic Fibrosis	5.48E-03	3.2 % 11/345

Top My Lists

Top My Pathways

(c) 2000-2024 QIAGEN. All rights reserved.

Name	p-value	Overlap
Network 2	6.74E-05	9.0 % 7/78

Top ML Disease Pathways

Name	p-value	Overlap
Hypoproteinemia	8.33E-04	12.1 % 4/33
Noninflammatory cervical disorder	1.16E-03	11.1 % 4/36
Cervical tumor	1.16E-03	11.1 % 4/36
Lesion of cervix	1.16E-03	11.1 % 4/36
Familial amyloidosis	3.98E-03	12.0 % 3/25

Top Analysis-Ready Molecules

Expr Fold Change

Molecules	Expr. Value	Chart
CCDC102B	↑ 86.656	
GPR179	↑ 57.180	
RIPK4	↑ 51.578	
EPHX2	↑ 39.652	
CYB5A	↑ 36.365	
FAM20A	↑ 34.336	

(c) 2000-2024 QIAGEN. All rights reserved.

INGENUITY PATHWAY ANALYSIS

**SYNGR2**	↑ 32.212
**ZNF618**	↑ 24.237
**AK3**	↑ 23.268
**SF3B4**	↑ 22.734

Expr Fold Change

Molecules	Expr. Value	Chart
**MBLAC2**	↓ -76.920	
**MFAP4**	↓ -62.500	
**SEC14L3**	↓ -58.824	
**RPL38**	↓ -55.556	
**LRPAP1**	↓ -47.619	
**FCN2**	↓ -45.455	
**CXCL5**	↓ -38.462	
**IGFBP7**	↓ -30.303	
**IGLV2-14**	↓ -26.316	
**ITGB1**	↓ -23.810	

(c) 2000-2024 QIAGEN. All rights reserved.

INGENUITY PATHWAY ANALYSIS

ID	Molecules in Network	Score	Focus Molecules	Top Diseases and Functions
1	A2M,ABCA13,ALB,APOC2,BLM,BPIFB1, C1QBP,CTNND1,CXCL5,CXCL6,CXCR2, CYP7A1,DNASE2,DNMT3A,DSC3,ELOC, ENPP2,ENPP3,EPO,FGF10,FYB1,GBP2, GCA,GMDS,GSPT1,HBB,HLA-DRA,HLA-DRB5, HLA-E,HSPD1,HSPG2,IFIT1,IGKC,IL13, IL15,IL1A,ITGB7,LBP,LILRA3,LRPAP1, MAEA,MAN2B2,MAP2K2,MAP3K1,MICA, MSLN,MUC1,MYDGF,NFATC3,OSCAR, PLPP3,PODXL,PRKCQ,PROC,PSMB8, PTGDR2,PTGES,RCC2,RHCG,RNASE2, RNASE4,SFRP1,Srgn,STIM1,SUPT16H, SYNGR2,TEK,TGM2,TNF,VEGFC	37	30	[Cellular Movement, Inflammatory Response, Organismal Injury and Abnormalities]
2	ACTB,ADAM17,ADRM1,ANGPT2,ANGPTL4, ARL6IP1,ATF6,CD1D,CD40LG,COL6A3,CRISPLD2, CTSB,DERL1,EIF2A,EIF2AK3,EPHX2,ERN1,F13A1 ,FCGBP,FGF10,FOXA1,FURIN,H1-2,HLA-DRB5,HSP90AA1, HSPD1,HTRA2,IGHG2,IGHG4, IL10RA,IL1RN,IL4,ITGAL,KLF6,KRT86, LPAR1,LSP1,LYPD3,MAFB,MT-ATP6,MYC, NMT1,NOS2,OSCAR,PFN1, PLA2G2A,PROC,PROCR,PSMA3,PSMB8, PTGDR2,RPL27A,RPL38,RPS19,RPTN, SELE,SPRR1A,STAT3,TGM2,TNFAIP6, TNFRSF17,TNFSF13,TPI1,TTR,TUBB, USP10,WFDC2,XBP1,ZAP70,ZNFX1	33	28	[Cell Death and Survival, Cellular Movement, Immune Cell Trafficking]
